# Model-estimated impacts of pediatric respiratory syncytial virus prevention programs in Mali on asthma prevalence

**DOI:** 10.1016/j.jacig.2023.100092

**Published:** 2023-05

**Authors:** Justin R. Ortiz, Rachel S. Laufer, Steven M. Brunwasser, Flanon Coulibaly, Fatoumata Diallo, Moussa Doumbia, Amanda J. Driscoll, Deshayne B. Fell, Fadima C. Haidara, Tina V. Hartert, Adama M. Keita, Kathleen M. Neuzil, Brittney M. Snyder, Samba Sow, Meagan C. Fitzpatrick

**Affiliations:** a University of Maryland School of Medicine, Baltimore, Md; b Center for Vaccine Development and Global Health, University of Maryland School of Medicine, Baltimore, Md; c Vanderbilt University Medical Center, Nashville, Tenn; d Rowan University, Glassboro, NJ; e Centre pour le Développement des Vaccins, Ministère de la Santé, Bamako, Mali; f School of Epidemiology and Public Health, University of Ottawa, and CHEO Research Institute, Ottawa, Ontario, Canada

**Keywords:** Respiratory syncytial virus (RSV), lower respiratory tract infection, immunoprophylaxis, asthma, global health, pediatrics, Mali

## Abstract

**Background:**

Respiratory syncytial virus (RSV) is a leading cause of lower respiratory tract infection (LRTI) in young children and is associated with subsequent recurrent wheezing illness and asthma (wheeze/asthma). RSV prevention may therefore reduce wheeze/asthma prevalence.

**Objectives:**

We estimated the contribution of RSV LRTI and the impact of RSV prevention on recurrent wheeze/asthma in Mali.

**Methods:**

We simulated 12 consecutive monthly birth cohorts in Mali and estimated RSV LRTI cases through 2 years and recurrent wheeze/asthma prevalence at 6 years under different RSV prevention scenarios: status quo, seasonal birth-dose extended half-life mAb, and seasonal birth-dose extended half-life mAb followed by 2 doses of pediatric vaccine (mAb + vaccine). We used World Health Organization (WHO) Preferred Product Characteristics for RSV prevention, demographic and RSV epidemiologic data from Mali, regional recurrent wheeze/asthma prevalence, and relative risk of recurrent wheeze/asthma given early childhood RSV LRTI.

**Results:**

Among the simulated cohort of 778,680 live births, 10.0% had RSV LRTI by 2 years and 89.6% survived to 6 years. We estimated that 13.4% of all recurrent wheeze/asthma at 6 years was attributable to RSV LRTI. Recurrent wheeze/asthma prevalence at 6 years was 145.0 per 10,000 persons (RSV LRTI attributable) and 1084.2 per 10,000 persons (total). In mAb and mAb + vaccine scenarios, RSV LRTI cases decreased by 11.8% and 44.4%, respectively, and recurrent wheeze/asthma prevalence decreased by 11.8% and 44.4% (RSV LRTI attributable) and 1.6% and 5.9% (total).

**Conclusion:**

In Mali, RSV prevention programs may have a meaningful impact on chronic respiratory disease, strengthening the case for investment in RSV prevention.

Respiratory syncytial virus (RSV) causes annual epidemics of acute respiratory infections in young children, and RSV lower respiratory tract infection (LRTI) is a major cause of morbidity and mortality in this age group.^1^, ^2^, ^3^, ^4^ Over 95% of all early childhood deaths from RSV LRTI are estimated to occur in low- and middle-income countries (LMICs).^1^^,^^4^ There is a licensed, short-acting RSV mAb, palivizumab, available for the prevention of RSV LRTI in high-risk infants; however, it is cost prohibitive for use by LMICs.^5^ Numerous RSV prevention candidates are in clinical development, including extended half-life mAbs, pediatric vaccines, and vaccines for use during pregnancy.^5^ If ultimately found to be safe and efficacious, each class could have an important role to play in preventing RSV LRTI and its sequalae in LMICs.

While prevention of RSV LRTI is the primary goal of RSV preventive interventions, impacts on additional clinical outcomes could enhance the public health value of these products.^3^^,^^6^ Early childhood RSV LRTI is associated with the subsequent development of recurrent wheezing illness and asthma (henceforth termed recurrent wheeze/asthma).^7^ Seeking evidence to inform expectations of potential additional impacts, public health consultations have called for studies of RSV prevention against chronic respiratory outcomes.^2^^,^^8^^,^^9^ Demonstrating impact on chronic respiratory outcomes in LMICs, in addition to the impact on RSV LRTI, could strengthen the case for investment in RSV prevention.^10^

The objectives of this modeling study were to estimate the contribution of RSV LRTI on recurrent childhood wheeze/asthma in Mali and to estimate the impact national RSV prevention programs would have on recurrent wheeze/asthma in Mali. We chose Mali for this study because it has excellent RSV epidemiologic data, it has a high prevalence of recurrent childhood wheezing with limited access to asthma care, and it is eligible for vaccine funding from Gavi, the Vaccine Alliance.

## Methods

### Study design

We simulated a 1-year birth cohort followed prospectively to estimate the plausible range of impacts of RSV LRTI illness, with and without preventive interventions, on recurrent wheeze/asthma among children in Mali. We adapted a health economic model that estimated the impact of RSV prevention on health care costs in Mali to assess the risk of subsequent recurrent wheeze/asthma after RSV LRTI illness.^11^^,^^12^ We used Mali-specific population, birthrate, and childhood mortality data to create 12 consecutive monthly birth cohorts.^13^^,^^14^

For the primary analysis, we modeled RSV LRTI clinical outcomes occurring during the first 2 years of life in 4 intervention scenarios. Briefly, they were (1) the baseline scenario (status quo) without any RSV prevention; (2) seasonal birth-dose immunoprophylaxis with extended half-life mAb; (3) seasonal birth-dose immunoprophylaxis with extended half-life mAb followed by 2 doses of RSV vaccine; and (4) a scenario analysis assuming that all RSV LRTI clinical outcomes were prevented to estimate the overall RSV LRTI–associated wheeze/asthma prevalence in Mali.

**Fig 1:**
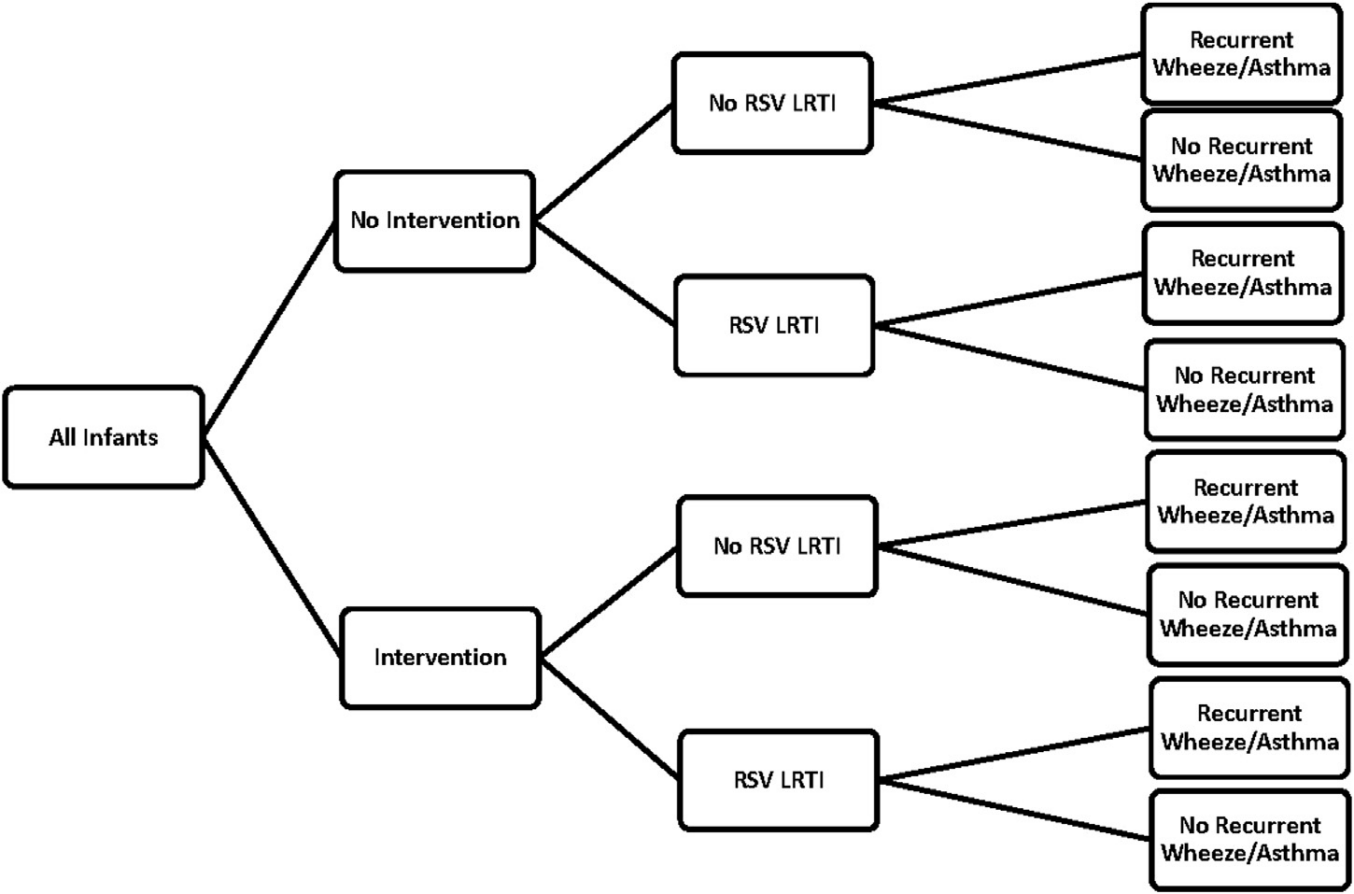
Probability outcome tree. Outcome tree model for simulated 12 consecutive monthly birth cohorts in Mali and estimated cumulative RSV LRTI cases through 2 years and recurrent wheeze/asthma prevalence at 6 years under different RSV prevention scenarios. Parameter inputs are detailed in Table I, and the analysis is described in the Methods. RSV LRTI depended on the probability of RSV illness and the probability of RSV LRTI given RSV illness, as well as characteristics of preventive intervention. RSV LRTI hospitalizations and deaths were simulated by incorporating probabilities of such events given RSV LRTI and are not included. The model was adapted from previous health economics analyses.^11^^,^^12^

We conducted secondary analyses to assess changes in key model assumptions. The scenarios and model inputs are detailed below and in Table I. The probability-based outcome tree is in Fig 1.

**Table I:** Description of model inputs

Parameter	Input used and rationale
RSV incidence rate	The overall community incidence rate of RSV in infants <6 months of age in Bamako, Mali, is estimated to be 536.8 per 1000 person years.^15^ Incidence varies by infant age and calendar month. Incidence rates for Mali were extrapolated out to 24 months based on the linear decline in the RSV incidence rates described for low-income countries between 6 and 24 months.^16^
RSV season	The RSV season is estimated to occur annually from July 1 through October 31, as determined by a community incidence study.^15^
Probability of LRTI given RSV illness	Among infants with confirmed RSV illness in Bamako, Mali, 29.4% (95% CI 23-37) had LRTI.^15^
Probability of inpatient care given RSV LRTI	We applied an age-specific gradient for hospitalization rates from the Gambia^1^ to the Mali-specific rate of hospitalization given RSV LRTI.^15^ Starting at 0.30 in the first month of life, these rates decline to 0.13 by the 24th month of life. In the Mali study, all infants with pneumonia who did not receive inpatient care received outpatient care. The probability of outpatient care given RSV LRTI is calculated as 1 minus the probability of inpatient care given RSV LRTI.
Case fatality rate among those who received inpatient care given RSV LRTI	We used age-specific hospital case fatality rates for RSV LRTI in LMICs,^15^^,^^16^ starting at 0.27 in the first month of life and declining to 0.009 by the 24th month of life. We assume 49% of RSV LRTI deaths occur outside the hospital.^1^
Baseline risk of recurrent childhood wheezing	We used Global Asthma Network–estimated prevalence of asthma symptoms among children aged 6 to 7 years in the African and Eastern Mediterranean region.^19^ For the primary analysis, we used 10.8% baseline risk of recurrent childhood wheezing. For sensitivity analyses, we used 4.3% prevalence of severe asthma symptoms estimated for African and Eastern Mediterranean region and 23.2% prevalence of current wheeze (highest single-center estimate).
RR of recurrent wheeze/asthma given early childhood RSV LRTI	We used a systematic review and meta-analysis that estimated an odds ratio from among studies that adjusted at least partially for genetic confounding; adjusted odds ratio was 2.45 (95% CI 1.23-4.88).^7^ For sensitivity analysis, we used the lower bound of the 95% CI, 1.23, as the RR estimate.
Crude birthrate	The crude birthrate for Mali in 2017 was estimated to be 42 per 1000 total population.^14^
Total country population	The total population for Mali in 2017 was estimated to be 18,540,000 persons.^14^
Number of infants in each monthly birth cohort	We calculated the number of infants in each monthly birth cohort by multiplying the crude birthrate by the total country population for Mali to estimate the number of infants born in 1 year. We then assigned the total number of births for each month based on the number of days in each month: 59,734 to 66,134.
Under 6 years childhood mortality	We used Mali estimates of under-5-year mortality (101 per 1000 children) and 5-to-9-year mortality (14.5 per 1000 children).^14^
RSV extended half-life mAb	We used a single birth dose before the RSV season. We assumed a single dose provided at birth to infants born during the RSV season or expected to enter any part of their first RSV season at <6 months of age.^11^^,^^12^ Efficacy was 70% and duration of protection was 5 months according to WHO Preferred Product Characteristics.^3^ We assumed 83% coverage based on birth-dose bacillus Calmette-Guérin vaccine coverage in Mali.^23^ We assumed that protection begins immediately after administration.
RSV vaccine	We used a vaccine provided as a 2-dose series in the Mali routine immunization schedule at 10 and 14 weeks. Efficacy was 70% and duration of protection was 12 months according to WHO Preferred Product Characteristics.^2^ We assumed 77% coverage based on DTP3 coverage in Mali.^23^ We assumed protection begins 2 weeks after the second dose. During the first 6 months of life, children receiving mAb + vaccine have fewer gaps in protection than children receiving mAb only, resulting in greater impact of the combined intervention.

*DTP3,* Third dose of diphtheria and tetanus toxoids and pertussis-containing vaccine.

### RSV epidemiologic inputs

We followed the simulated cohort from birth through 24 months and estimated RSV LRTI episodes, RSV LRTI hospitalizations, and RSV LRTI deaths during that time. For the first 6 months of life, we used month- and age-specific community-based RSV incidence rates per 1000 child years in Mali.^15^ We extrapolated incidence rates out to 24 months based on the linear decline in the RSV incidence rates described for low-income countries between 6 and 24 months.^16^ We stopped modeling RSV infection after 24 months, as the anticipated duration of protection of the RSV LRTI prevention programs would not extend beyond this age. Probabilities of illness depended on Mali-specific RSV LRTI incidence rates given RSV illness.^12^ The RSV season was July 1 through October 31.^15^ We used age-specific rates of hospitalization given the RSV LRTI and RSV LRTI–attributable mortality among infants hospitalized with RSV LRTI in LMICs.^17^ We made the conservative assumption that previous RSV illness protected children from subsequent RSV LRTI outcomes.^18^

### Recurrent wheeze/asthma epidemiologic inputs

Wheeze-associated disorders are a common cause of morbidity in children.^19^ Asthma is a cause of recurrent wheezing, but it cannot easily be objectively diagnosed using lung function tests until about 6 years of age.^20^ Global estimates rely on wheezing symptom surveys. For the baseline prevalence of recurrent wheeze/asthma at 6 years of age, we used Global Asthma Network estimates.^19^ In 2021, the Global Asthma Network reported global and regional estimates of symptoms of asthma among children aged 6 to 7 years. The Global Asthma Network instruments define “current wheeze” as wheezing or whistling in the chest reported in the past 12 months, and “severe asthma symptoms” as current wheeze in addition to 4 or more attacks of wheeze, 1 or more nights per week with sleep disturbance from wheeze, or wheeze affecting a child’s speech.^19^ For the primary analyses, we assumed the baseline (all-cause) prevalence of recurrent wheeze/asthma to be 10.8%, which is the Global Asthma Network estimate for the African and Eastern Mediterranean region.^19^

The relative risk (RR) of recurrent wheeze/asthma given early childhood RSV LRTI was drawn from a 2020 systematic review and meta-analysis.^7^ The review included observational studies of the risk of subsequent recurrent wheeze/asthma among children with medically attended RSV LRTI in the first 2 years of life. The meta-analysis partially adjusted for inherited predisposition to recurrent wheezing illness.^7^ For the primary analysis, we used the pooled adjusted odds ratio of 2.45 to estimate the RR of the subsequent development of recurrent wheeze/asthma at 6 years of age among children with previous RSV LRTI compared to those with no RSV LRTI. Given the low prevalence of recurrent wheeze/asthma among those without RSV LRTI in our primary analysis, the odds ratio can be used to estimate the RR.^21^

To estimate the prevalence of recurrent wheeze/asthma, we first determined the number of children surviving to 6 years of age by applying childhood mortality estimates for Mali to the number of live births in the simulated birth cohort.^13^^,^^14^ We calculated the number of surviving RSV LRTI cases similarly. Because the Global Asthma Network prevalence estimates are for populations with and without prior RSV LRTI, we calculated the prevalence of recurrent wheeze/asthma among children without RSV LRTI (Prevalence_NoRSV_) in the status quo analysis using the following equation (with illustrative inputs from the primary status quo analysis):

$${Prevalence}_{NoRSV}=\left({Prevalence}_{Total}\times {Population}_{Total}\right)/\left[\left({RR}_{Wheeze}\times {Population}_{RSV}\right)+{Population}_{NoRSV})\right]$$

where Prevalence_Total_ is the all-cause overall prevalence of asthma in the population (10.8%); Population_Total_ is the total population of children surviving to 6 years of age (698,003); RR_Wheeze_ is the estimated RR of recurrent wheeze/asthma given early childhood RSV LRTI (2.45); Population_RSV_ is the number of children with RSV LRTI surviving to 6 years of age (74,331); and Population_NoRSV_ is the number of children without RSV LRTI surviving to 6 years of age (623,673). We used Prevalence_NoRSV_ as the baseline for recurrent wheeze/asthma in our model. For the primary analyses, Prevalence_NoRSV_ was calculated to be 9.4%.

We computed the all-cause, total recurrent wheeze/asthma prevalence at 6 years of age as the sum of prevalent cases among children with and without a history of RSV LRTI, expressed as a proportion of the total population of children surviving to 6 years of age. To calculate the prevalent cases among children with RSV LRTI in the primary intervention analyses, we multiplied the number of children with RSV LRTI and surviving to 6 years of age per prevention scenario by Prevalence_NoRSV_ and by RR_Wheeze_. To calculate the prevalent cases among children without RSV LRTI, we multiplied the number of children not experiencing RSV LRTI and surviving to 6 years of age per prevention scenario by Prevalence_NoRSV_. We calculated the number of RSV LRTI–attributable cases according to the following equation:

$$RSV\;LRTI-attributable\;recurrent\;wheeze/asthma\;cases=\left[\left({Prevalence}_{NoRSV}\times {Population}_{NoRSV}\times {RR}_{Wheeze}\right)-\left({Prevalence}_{NoRSV}\times {Population}_{NoRSV}\right)\right]$$

The number of RSV LRTI–attributable recurrent wheeze/asthma cases was the difference of the calculated prevalence among the subset of children with RSV LRTI applying baseline prevalence among children without RSV LRTI (Prevalence_NoRSV_) and RR increase given early childhood RSV LRTI (RR_Wheeze_), minus the calculated cases among the same subset applying baseline prevalence (Prevalence_NoRSV_) only.

### Interventions

**Fig 2:**
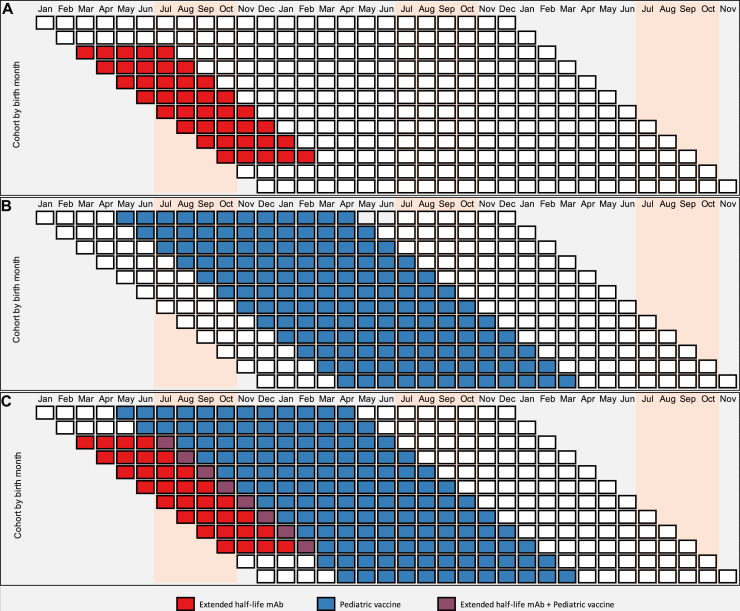
Timing and duration of RSV LRTI prevention strategies for the simulated monthly birth cohorts. **(A)** Twelve-month birth cohort, represented by different rows of boxes beginning under the birth month, RSV season *(peach),* and mAb protection period *(red).* Shown is the extended half-life mAb intervention scenario. **(B)** Twelve-month birth cohort and RSV season, shown similarly with pediatric vaccine protection period. A pediatric vaccine-only scenario is not simulated in the study, but it is included to illustrate the period of pediatric vaccine-only protection. **(C)** Twelve-month birth cohort and RSV season with the mAb + pediatric vaccine protection period, showing the extended half-life mAb + vaccine intervention scenario.

For the primary analyses, we assessed the impacts of different prevention program scenarios. For the first scenario, we assessed no intervention (status quo), which provided a baseline for subsequent intervention impact estimates. For the second scenario, we used a single dose of extended half-life mAb followed by 2 doses of RSV vaccine during routine childhood immunization visits. We applied WHO Preferred Product Characteristics for RSV mAbs for intervention characteristics.^3^ Seasonal birth-dose immunoprophylaxis was provided as a single dose provided at birth to infants born during the RSV season or expected to enter their first RSV season at less than 6 months of age, with 70% efficacy and durability of 5 months.^3^ We assumed that extended half-life mAb protection began at the time of administration. These inputs are aligned with clinical data on a leading extended half-life mAb candidate.^22^ Intervention coverage used 2019 Mali estimates for birth-dose bacillus Calmette-Guérin vaccine (83%).^23^ While RSV vaccines for use during pregnancy are under development and have the same preferred efficacy by the WHO,^2^ their development lags behind mAbs, and they are anticipated to provide a shorter duration of infant protection.^3^^,^^24^ This scenario provides estimates for realistic intervention impact in the near term. For the third scenario, we used a single dose of extended half-life mAb as described above, followed by 2 doses of RSV vaccine during routine childhood immunization visits (extended half-life mAb + vaccine). We applied WHO Preferred Product Characteristics for the pediatric RSV vaccines.^2^ We assumed a 2-dose series for pediatric RSV vaccines, administered to infants at established 10- and 14-week routine immunization visits, with 70% efficacy and durability of 12 months. We assumed pediatric vaccine protection began 2 weeks after the second dose. Intervention coverage used 2019 Mali estimates for third-dose diphtheria, tetanus, and pertussis vaccine (77%).^23^ Pediatric RSV vaccines are not as advanced in clinical development as mAbs or RSV vaccines for use during pregnancy.^24^ Our goal with this second scenario was to illustrate what the maximum, feasible ceiling of wheeze/asthma prevention would be in Mali. Aligned with WHO Preferred Product Characteristics, each product (extended half-life mAb and pediatric vaccines) was assumed to prevent RSV LRTI and not RSV infection.^2^^,^^3^ For the fourth scenario, we assessed the theoretical elimination all RSV LRTI from Mali, which allowed us to estimate the overall RSV LRTI–associated wheeze/asthma in the country. All scenario analyses were identical to the status quo analysis, except for the probability of RSV LRTI. Fig 2 shows the timing and duration of RSV LRTI protection for the prevention strategies.

### Uncertainty and secondary analyses

We integrated empirical parameter uncertainty into our primary analysis using a Monte Carlo approach with 10,000 independent trials. We randomly sampled 1 value from each empirical distribution of probabilities for each trial and calculated the clinical outcomes associated with the status quo and prevention scenarios. For estimates of RSV LRTI and recurrent wheeze/asthma outcomes, we defined 95% confidence intervals (CIs) as the range encompassing 95% of the values produced across all trials.

We conducted secondary analyses to assess the sensitivity of our conclusions to changes in 3 model assumptions, holding all other model elements constant, as follows: model 1, Global Asthma Network–estimated prevalence of severe asthma symptoms in the past 12 months for the African and Eastern Mediterranean region (5.5%);^19^ model 2, Global Asthma Network–estimated prevalence of recurrent wheeze/asthma equal to the highest single-center prevalence for current wheeze reported from Costa Rica (23.2%);^19^ and model 3, RR of recurrent wheeze/asthma given early childhood RSV LRTI equal to the lower bound of the 95% CI for the pooled estimate from the published meta-analysis (1.23).^7^

We tabulated the number of RSV LRTI outcomes for the birth cohort by year of life and overall for the intervention scenarios. Next, we tabulated the RSV LRTI–attributable recurrent wheeze/asthma prevalence and the total recurrent wheeze/asthma prevalence by 6 years of age for the intervention scenarios. We calculated the percentage change between intervention scenarios from the status quo. Because our estimates of product efficacy and coverage were point estimates without CIs, 95% CIs around the percent change estimates were not meaningful and thus were not calculated. We performed all analyses by R v4.0.2 software (R Project; www.r-project.org). Because there were no human subjects or personally identifiable information, institutional review board approval was not required.

### Role of the funding source

Bill & Melinda Gates Foundation funded this study. The funder had no role in the study design, study conduct, report writing, or decision to submit the manuscript for publication.

## Results

### Study population

In our model, the total population of Mali was 18,540,000 persons. The simulated birth cohort included 778,680 live births in 1 year, with an average of 64,890 live births per month. Of these live births, we estimated that 89.6% (698,003/778,680) lived to 6 years of age.

### Status quo scenario

**Fig 3:**
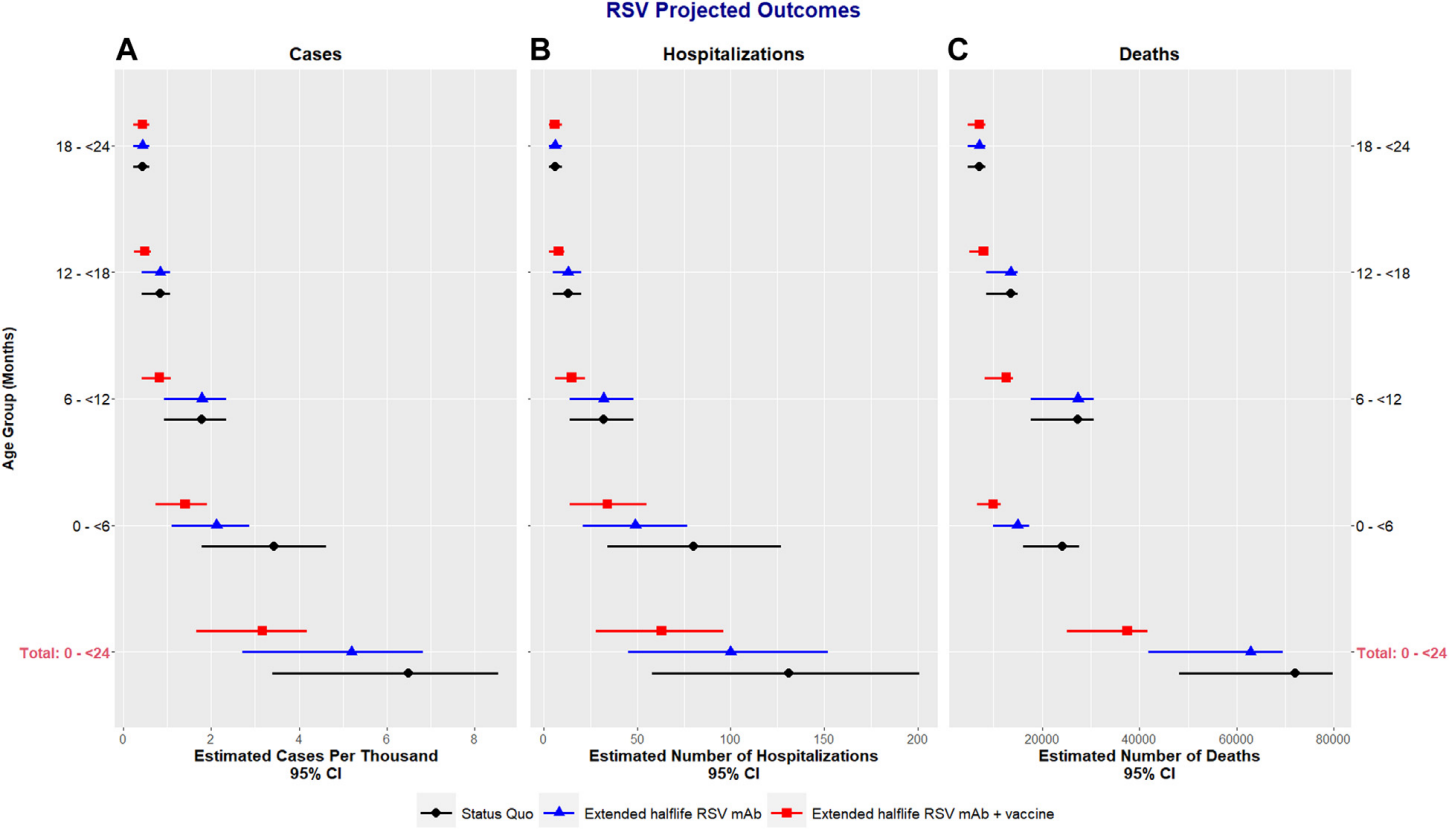
RSV LRTI outcomes in Mali by intervention and age group.

**Fig 4:**
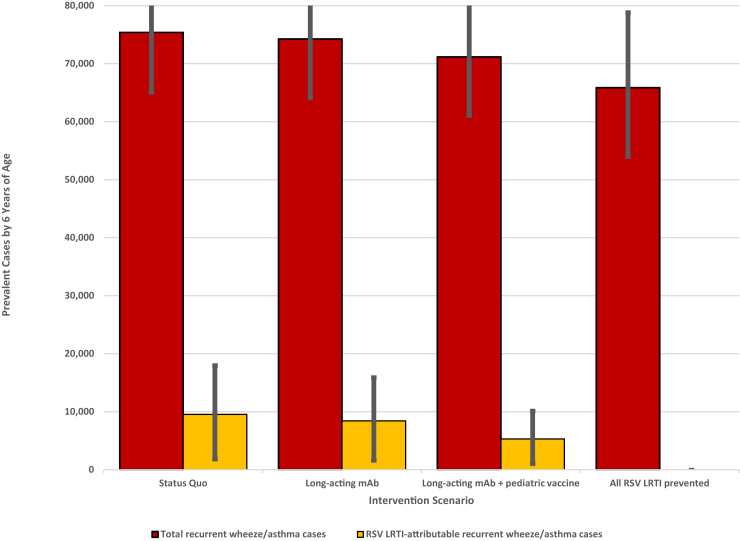
RSV-attributable and total recurrent wheeze/asthma cases at 6 years of age in Mali by intervention.

Without national RSV prevention programs (ie, status quo scenario), we estimated that the birth cohort had 71,984 (95% CI 48,113-79,781) RSV LRTI episodes, 6494 (95% CI 3402-8536) RSV LRTI hospitalizations, and 131 (95% CI 58-201) RSV LRTI deaths from birth through 2 years of age (Table II and Fig 3). Within the first 6 months of life, we estimated 24,087 (95% CI 16,054-27,697) RSV LRTI, 3423 (95% CI 1782-4614) RSV LRTI hospitalizations, and 80 (95% CI 34-127) RSV LRTI deaths occurred. Overall, we estimated that 75,680 (95% CI 65,312-86,640) of the surviving children in the birth cohort had recurrent wheeze/asthma at 6 years of age (Table III and Fig 4). In 13.4% (95% CI 3.3-24.4) of these children, the recurrent wheeze/asthma was attributable to early life RSV LRTI. The cause-specific prevalence of RSV LRTI–attributable recurrent wheeze/asthma was 145.0 per 10,000 children (95% CI 34.9-265.6), and the all-cause, total prevalence of recurrent wheeze/asthma was 1084.2 per 10,000 children (95% CI 935.7-1241.3). Had all RSV LRTI in the cohort been prevented, the prevalence of recurrent wheeze/asthma would have been 939.2 per 10,000 children (95% CI 769.7-1124.5).

**Table II:** Modeled RSV LRTI outcomes through 2 years of life in Mali by intervention and age group

Intervention	Age (months)	RSV LRTI cases		RSV LRTI hospitalizations		RSV LRTI deaths	
		Frequency (95% CI)	Decrease from status quo	Frequency (95% CI)	Decrease from status quo	Frequency (95% CI)	Decrease from status quo
Status quo	0 to <6	24,087 (16,054-27,697)	—	3423 (1782-4614)	—	80 (34-127)	—
	6 to <12	27,359 (17,725-30,552)	—	1794 (923-2345)	—	32 (14-48)	—
	12 to <18	13,488 (8,524-14,937)	—	839 (419-1077)	—	13 (5-20)	—
	18 to <24	7050 (4685-8397)	—	438 (229-603)	—	6 (3-10)	—
	Total	71,984 (48,113-79,781)	—	6494 (3402-8536)	—	131 (58-201)	—
Extended half-life RSV mAb	0 to <6	14,936 (9848-17,300)	38.0%	2122 (1100-2865)	38.0%	49 (21-77)	38.8%
	6 to <12	27,359 (17,724-30,552)	0%	1794 (923-2345)	0%	32 (14-48)	0%
	12 to <18	13,488 (8524-14,937)	0%	839 (419-1077)	0%	13 (5-20)	0%
	18 to <24	7050 (4685-8397)	0%	438 (229-603)	0%	6 (3-10)	0%
	Total	62,833 (41,905-69,504)	20.0%	5193 (2720-6819)	20.0%	100 (45-152)	23.7%
Extended half-life RSV mAb + vaccine	0 to <6	9924 (6579-11,446)	58.8%	1410 (733-1907)	58.8%	34 (14-55)	57.5%
	6 to <12	12,612 (8171-14,084)	53.9%	827 (426-1081)	53.9%	15 (6-22)	53.9%
	12 to <18	7935 (5018-8805)	41.2%	494 (246-638)	41.1%	8 (3-11)	38.5%
	18 to <24	7050 (4685-8397)	0%	438 (229-603)	0%	6 (3-10)	0%
	Total	37,521 (25,115-41,659)	47.9%	3169 (1663-4174)	51.2%	63 (28-96)	51.9%

We simulated 12 consecutive monthly birth cohorts in Mali, estimated cumulative RSV LRTI cases through 2 years, and evaluated the impact of different RSV prevention scenarios: status quo, birth-dose seasonal extended half-life RSV mAb, and birth-dose seasonal extended half-life mAb followed by 2 doses of pediatric vaccine. We used WHO Preferred Product Characteristics for RSV prevention, demographic and RSV epidemiologic data from Mali, regional recurrent wheeze/asthma prevalence, and RR of recurrent wheeze/asthma given early childhood RSV LRTI. Extended half-life RSV mAb will only provide protection during the first 6 months of life, while extended half-life RSV mAb + pediatric vaccine will provide protection into the 12 to <18 month age group. Because the proposed causal mechanisms for RSV LRTI–attributable recurrent wheeze and asthma occur before 2 years of age, we stopped modeling RSV infection after 24 months. The 95% CIs were not calculated for percentage decrease because product efficacy and coverage are both specified as point estimates.

**Table III:** RSV-attributable and total recurrent wheeze/asthma prevalence at 6 years of age in Mali by intervention

Intervention	RSV LRTI–attributable recurrent wheeze/asthma			Total recurrent wheeze/asthma		
	Cases at 6 years (95% CI)	Prevalence per 10,000 children at 6 years (95% CI)	Percentage decrease from status quo (95% CI)	Cases at 6 years (95% CI)	Prevalence per 10,000 children at 6 years (95% CI)	Percentage decrease from status quo (95% CI)
Status quo	10,123 (2436-18,541)	145.0 (34.9-265.6)	—	75,680 (65,312-86,640)	1084.2 (935.7-1241.3)	—
Extended half-life mAb	8930 (2148-16,388)	127.9 (30.8-234.8)	11.8% (10.4-13.5)	74,487 (64,275-85,427)	1067.1 (920.8-1223.9)	1.6% (0.4-2.9)
Extended half-life mAb + vaccine	5632 (1368-10,434)	80.7 (19.6-149.5)	44.4% (42.5-45.5)	71,189 (61,069-82,593)	1019.9 (874.9-1183.3)	5.9% (1.4-10.8)
All RSV LRTI prevented∗	0	0	100.0%	65,557 (53,722-78,489)	939.2 (769.7-1124.5)	13.4% (3.3-24.4)

Analysis assumed a causal association between early childhood RSV LRTI and subsequent development of recurrent wheeze/asthma. We simulated 12 consecutive monthly birth cohorts in Mali and estimated cumulative RSV LRTI cases through 2 years and recurrent wheeze/asthma prevalence at 6 years under different RSV prevention scenarios: status quo, birth-dose seasonal extended half-life mAb, and birth-dose seasonal extended half-life mAb followed by 2 doses of pediatric vaccine (mAb + vaccine). We used WHO Preferred Product Characteristics for RSV prevention, demographic and RSV epidemiologic data from Mali, regional recurrent wheeze/asthma prevalence, and RR of recurrent wheeze/asthma given early childhood RSV LRTI. RSV LRTI–attributable wheeze/asthma cases are calculated among all children experiencing RSV LRTI. The number of RSV LRTI–attributable recurrent wheeze/asthma cases was the difference of the calculated prevalence among the subset of children with RSV LRTI applying baseline prevalence without previous RSV LRTI (9.4%) and RR increase (2.45) minus the calculated cases among the same subset applying baseline prevalence without previous RSV LRTI (9.4%) only. Total wheeze/asthma cases are the sum of (1) the calculated prevalence applying baseline prevalence without previous RSV LRTI (9.4%) among all children without history of RSV LRTI plus (2) the calculated prevalence applying baseline prevalence without previous RSV LRTI (9.4%) and RR (2.45-fold) increase due to early childhood RSV LRTI among all children with a history of RSV LRTI.

∗ This scenario assumed 100% RSV LRTI prevented, so we did not calculate 95% CI for RSV LRTI–attributable outcomes.

### Prevention scenarios

Extended half-life mAb programs decreased RSV LRTI by 20.0%, RSV LRTI hospitalizations by 20.0%, and RSV LRTI deaths by 23.7% compared to status quo in the first 2 years of life (Table II and Fig 3). The greatest benefits were achieved in the first 6 months of life, in which the intervention reduced RSV LRTI by 38.0%, RSV LRTI hospitalizations by 38.0%, and RSV LRTI deaths by 38.8% from the status quo. In the context of national extended half-life mAb programs, children surviving to 6 years of age would have a cause-specific prevalence of RSV LRTI–attributable recurrent wheeze/asthma of 127.9 per 10,000 children (95% CI 30.8-234.8) and an all-cause total prevalence of recurrent wheeze/asthma of 1067.1 per 10,000 children (95% CI 920.8-1223.9) (Table III and Fig 4). Compared to the status quo, extended half-life mAb decreased the prevalence of recurrent wheeze/asthma by 11.8% for RSV LRTI–attributable cases and 1.6% overall.

Extended half-life mAb + vaccine programs decreased RSV LRTI by 47.9%, RSV LRTI hospitalizations by 51.2%, and RSV LRTI deaths by 51.9% compared to the status quo in the first 2 years of life (Table II and Fig 3). In this scenario, RSV LRTI prevention was sustained into the 12 to <18 month age group. Children surviving to 6 years of age had a prevalence of RSV LRTI–attributable recurrent wheeze/asthma of 80.7 per 10,000 children (95% CI 19.6-149.5) and a prevalence of total recurrent wheeze/asthma of 1019.9 per 10,000 children (95% CI 874.9-1183.3) (Table III and Fig 4). Compared to the status quo, this scenario decreased the prevalence of recurrent wheeze/asthma by 44.4% for RSV LRTI–attributable cases and 5.9% overall.

### Secondary analyses

Changes in assumptions of the baseline of recurrent wheeze/asthma did not change the relative decrease in cases by interventions, but they did change the absolute reduction in cases (Table IV). When we used the 5.5% baseline of severe asthma symptoms in the model, we estimated extended half-life mAb reduced the absolute number of such cases by 605 (1.6% decrease from status quo). Extended half-life mAb + vaccine reduced cases of severe asthma symptoms by 2278 (5.9% decrease from status quo). A higher baseline prevalence assumption of 23.2% resulted in a greater absolute reduction in cases but the same relative decreases for each intervention.

**Table IV:** Secondary analyses of RSV-attributable and total recurrent wheeze or asthma prevalence at 6 years of age in Mali by intervention and alternative assumptions

Scenario	RSV LRTI–attributable recurrent wheeze/asthma			Total recurrent wheeze/asthma		
	No. of cases at 6 years	Prevalence per 10,000 children at 6 years	Decrease from status quo	No. of cases at 6 years	Prevalence per 10,000 children at 6 years	Decrease from status quo
Severe asthma symptoms in past 12 months for African and Eastern Mediterranean region (5.5%)∗						
Status quo	5135	73.6	—	38,390	550.0	—
Extended half-life mAb	4530	64.9	11.8%	37,785	541.3	1.6%
Extended half-life mAb + vaccine	2857	40.9	44.4%	36,112	517.4	5.9%
All RSV LRTI prevented	0	0.0	100.0%	33,255	476.4	13.4%
Highest center recurrent wheeze/asthma prevalence estimate (23.2%)∗						
Status quo	21,660	310.3	—	161,937	2320.0	—
Extended half-life mAb	19,109	273.8	11.8%	159,385	2283.4	1.6%
Extended half-life mAb + vaccine	12,052	172.7	44.4%	152,328	2182.3	5.9%
All RSV LRTI prevented	0	0.0	100.0%	140,276	2009.7	13.4%
Low RR of recurrent wheeze/asthma given early childhood RSV LRTI (RR = 1.23)†						
Status quo	1809	25.9	—	75,680	1084.2	—
Extended half-life mAb	1596	22.9	11.8%	75,466	1081.2	0.3%
Extended half-life mAb + vaccine	1007	14.4	44.4%	74,877	1072.7	1.1%
All RSV LRTI prevented	0	0.0	100.0%	73,870	1058.3	2.4%

Analysis assumed a causal association between early childhood RSV LRTI and subsequent development of recurrent wheeze/asthma. We simulated 12 consecutive monthly birth cohorts in Mali and estimated cumulative RSV LRTI cases through 2 years and recurrent wheeze/asthma prevalence at 6 years under different RSV prevention scenarios: status quo, birth-dose seasonal extended half-life mAb, and birth-dose seasonal extended half-life mAb followed by 2 doses of pediatric vaccine (mAb + vaccine). We used WHO Preferred Product Characteristics for RSV prevention, demographic and RSV epidemiologic data from Mali, regional recurrent wheeze/asthma prevalence, and RR of recurrent wheeze/asthma given early childhood RSV LRTI. RSV LRTI–attributable wheeze or asthma cases are calculated among all children experiencing RSV LRTI. The number of RSV LRTI–attributable recurrent wheeze/asthma cases was the difference of the calculated prevalence among the subset of children with RSV LRTI applying baseline prevalence without previous RSV LRTI and RR increase minus the calculated cases among the same subset applying baseline prevalence without previous RSV LRTI only. Total wheeze or asthma cases are the sum of (1) among all children without history of RSV LRTI, the calculated prevalence applying baseline prevalence without previous RSV LRTI plus (2) among all children with a history of RSV LRTI, the calculated prevalence applying baseline prevalence without previous RSV LRTI and RR increase due to early childhood RSV LRTI.

∗ For the primary analyses, we used overall recurrent wheeze/asthma estimates of 10.8% (African region). For secondary analyses, we used 5.5% (severe asthma symptoms in the past 12 months for the African and Eastern Mediterranean region) and 23.2% (highest single-center estimate).^19^ We then calculated the baseline prevalence without previous RSV LRTI, as discussed in the Methods.

† For RR of recurrent wheeze/asthma given early childhood RSV LRTI, we used 2.45 for the primary analyses, and we used 1.23 for this sensitivity analysis (1.23 was the lower bound of the 95% CI from the pooled estimate in a recent meta-analysis^7^).

We also explored the impact of a lower estimate of RR of developing recurrent wheeze/asthma given early childhood RSV LRTI, using a RR of 1.23 (the lower bound of the 95% CI estimate from the meta-analysis).^7^ In this analysis, the maximum percentage decrease from the status quo that could be accomplished by extended half-life mAb was 0.3% and by prevention of all RSV LRTI illness was 2.4%.

### Conclusions

RSV is the greatest contributor to LRTI among young children globally.^4^ Our study suggests that it may also be a major contributor to pediatric asthma. We estimate that 13.4% of all recurrent wheeze/asthma among children aged 6 years in Mali may be attributable to RSV LRTI. The benefits of RSV prevention may therefore extend beyond reducing early childhood LRTI and include clinically meaningful impacts on chronic lung disease prevalence. The Global Asthma Network estimates that West Africa has high pediatric asthma and severe asthma prevalence.^19^ Because high-quality asthma care is limited in Mali,^25^ asthma-preventive interventions would have high public health value.

In this study incorporating pre–coronavirus disease 2019 pandemic data, we estimated that routine extended half-life RSV mAb programs can be expected to reduce RSV LRTI cases, hospitalizations, and deaths by around 20% in Mali. Because only a minority of children experience RSV LRTI, we anticipate that RSV mAb prevention programs would reduce pediatric asthma prevalence by 1.6%. Programs combining extended half-life RSV mAb with pediatric RSV vaccines could reduce about 50% of RSV LRTI cases, hospitalizations, and deaths, and would decrease pediatric asthma prevalence by 5.9%. The coronavirus disease 2019 pandemic is associated with alterations of the seasonality and circulation of many respiratory viruses, including RSV. It remains to be seen whether RSV activity will return to prepandemic baselines.

A 2021 cost-effectiveness analysis concluded that future RSV vaccines and mAbs may be cost-effective in Mali, although at higher costs per illness averted than most other public health interventions deployed in the country.^12^ This health economic analysis only evaluated RSV LRTI outcomes. Our study highlights that the full public health value of RSV LRTI prevention may be greater. Furthermore, RSV LRTI prevention may have additional impacts on asthma that we did not evaluate, including potential beneficial effects on the developing immune system, the airway microbiome, and the developing airway epithelium.^26^, ^27^, ^28^ These are likely to modify the risk of subsequent infections, such as rhinovirus, the most common cause of early childhood recurrent wheezing.^28^ In addition, many children with RSV LRTI will receive antibiotics, which in turn may increase asthma risk.^29^

Our analysis assumed a causal effect of RSV LRTI on subsequent recurrent wheeze/asthma; however, whether there is a causal relationship between RSV LRTI and asthma has not been firmly established.^6^^,^^7^^,^^30^^,^^31^ An extensive systematic review and meta-analysis exploring this association found that studies that partially controlled for genetic influences produced associations that were lower in magnitude than studies that did not.^7^ The review concluded that this was consistent with what would be expected if RSV LRTI were at least partly a marker of genetic susceptibility rather than a purely causal factor and that the calculated pooled odds ratio (2.45) may be overestimated as a result of residual confounding by shared heredity.^7^ While the review pooled analyses that included children across a range of gestational ages as well as outpatient and inpatient RSV LRTI outcomes, it did not assess gestational age–specific or RSV LRTI severity-specific risks. We could not adjust by these variables in our analysis.^7^ If the RR of recurrent wheeze/asthma given early childhood RSV LRTI is in fact lower, then the potential for RSV immunoprophylaxis to meaningfully affect this outcome would be reduced, as shown by our sensitivity analysis, where a RR of 1.23 corresponded to only 2.4% of total recurrent wheeze/asthma being attributable to RSV LRTI.

Our study should be interpreted in the context of its limitations. First, we relied on global systematic reviews/meta-analyses for assumptions about baseline risk and effect of RSV LRTI on recurrent wheeze/asthma risk in Mali. Next, our static model could not estimate potential herd effects of RSV prevention, although we believe that preventive interventions targeting infant RSV LRTI are unlikely to result in substantial herd immunity. We did not include differential risk of RSV LRTI–caused recurrent wheeze/asthma through the first 2 years of life; however, there is evidence to suggest that LRTI earlier in life results in more detrimental airway and immunologic changes.^28^ If this were the case, we may have underestimated the impact of prevention on chronic respiratory outcomes. We assumed RSV illness protects from future RSV infection; however, a 2021 Canadian cohort study of over 10,000 children estimated that 6.5% of infants hospitalized with RSV LRTI had reinfections.^18^ If this were true in Mali, the total RSV LRTI cases and RSV LRTI–attributable recurrent wheeze/asthma prevalence would decrease by 6.5%. Next, the RSV preventive intervention characteristics used in our models reflected WHO Preferred Product Characteristics; while early-phase controlled trials indicate mAb characteristics may be similar to our assumptions,^22^ it may be challenging for pediatric vaccines provided at 10 and 14 weeks to meet 70% efficacy.^5^ Finally, we excluded RSV vaccination during pregnancy, which WHO Preferred Product Characteristics indicate may have shorter duration of protection compared to mAbs,^2^ and which is likely to have lower coverage, given limitations in the maternal immunization platform in Mali.^12^^,^^23^ We also excluded other interventions that WHO did not consider in the Preferred Product Characteristics, such as palivizumab provided monthly to high-risk infants or extended half-life mAb given every 6 months through infancy.

Our study highlights that a single dose of RSV immunoprophylaxis provided at birth within existing immunization platforms can substantially impact RSV LRTI illness. Additional effects on the prevalence of chronic respiratory outcomes would enhance the value proposition of RSV prevention programs. Public health decision makers should keep these and other potential indirect benefits in mind when making RSV prevention funding and implementation recommendations.

**Key messages**

• RSV LRTI is associated with recurrent wheeze/asthma.
• Approximately 13.4% of all recurrent wheeze/asthma may be attributable to RSV LRTI in Mali.
